# ﻿Morpho-phylogenetic evidence reveals novel Bambusicolous fungi from Guizhou Province, China

**DOI:** 10.3897/mycokeys.118.149455

**Published:** 2025-06-02

**Authors:** Yao Feng, Ya-Ya Chen, Chuan-Gen Lin, Zuo-Yi Liu, Xiao-Fang Chen, Guo-Shun Pei, Jian-Kui Liu

**Affiliations:** 1 School of Chinese Ethnic Medicine, Guizhou Minzu University, Guiyang, Guizhou 550006, China; 2 Guizhou Key Laboratory of Agricultural Biotechnology, Guizhou Academy of Agricultural Sciences, Guiyang, Guizhou 550006, China; 3 Institute of Crop Germplasm Resources, Guizhou Academy of Agricultural Sciences, Guiyang, Guizhou 550025, China; 4 School of Life Sciences, Guizhou Normal University, Guiyang, Guizhou 550025, China; 5 Bijie Medical College, Bijie, Guizhou 551700, China; 6 School of Life Science and Technology, Center for Informational Biology, University of Electronic Science and Technology of China, Chengdu, Sichuan 611731, China

**Keywords:** 3 new taxa, Bambusicolaceae, multi-gene, phylogeny, taxonomy

## Abstract

The diversity and ecological importance of fungi associated with bamboo ecosystems are increasingly recognized, as these fungi play crucial roles in nutrient cycling and bamboo decomposition. This study identified five ascomycetous species from decaying bamboo stems in Guizhou, China. Phylogenetic analyses based on a concatenated dataset of SSU, ITS, LSU, *rpb2*, and *tef1-α* sequences confirmed their placement within the family Bambusicolaceae. Three new species, *Bambusicolapseudodimorphae*, *Bambusicolagelatinosospora*, and *Bambusicolaellipsospora*, are described as new to science, while two previously known species, *Bambusicolaautumnalis* and *Corylicolaitalica*, are also reported. Notably, this study provides the first account of the asexual morph of *Bambusicolaautumnalis*, and *Corylicolaitalica* is newly recorded from China. Comprehensive morphological descriptions, illustrations, and phylogenetic assessments are presented, contributing to the expanding knowledge of fungi. These findings underscore the importance of continued exploration of fungal diversity in bamboo ecosystems.

## ﻿Introduction

Bambusoideae, a subfamily of Poaceae, exhibits remarkable species diversity (Shi et al. 2020). As a dominant group within this subfamily, bamboos provide substantial economic, ecological, and social benefits, making them an essential resource in modern forestry and regional economies (Lobovikov et al. 2007; Shukla et al. 2016). Fungi associated with bamboo, collectively called bambusicolous fungi, inhabit various bamboo tissues (Hino 1938; Hyde et al. 2002). These fungi include pathogens that compromise bamboo health (Kuai 1996), endophytes that promote growth and enhance stress resistance (Yan et al. 2023), and saprobes that facilitate organic matter decomposition, nutrient cycling, and energy transformation within bamboo ecosystems (Senanayake et al. 2020; Feng et al. 2021; Jiang et al. 2021, 2022; Liang et al. 2023; Zhang et al. 2023; Yu et al. 2024). With increasing research on bambusicolous fungi, numerous new species continue to be reported (Kwon et al. 2022; Jiang et al. 2022; Yang et al. 2023; Yu et al. 2024).

The family Bambusicolaceae was established by Hyde et al. (2013) to accommodate the type genus *Bambusicola* based on comprehensive morphological and molecular evidence. Subsequent studies expanded the family to include additional genera, such as *Leucaenicola*, *Longipedicellata*, *Neobambusicola*, and *Palmiascoma* (Crous et al. 2014; Liu et al. 2015; Jayasiri et al. 2019). However, *Neobambusicola* and *Longipedicellata* were later transferred to Sulcatisporaceae and Longipedicellataceae, respectively, following detailed morphological and phylogenetic analysis (Tanaka et al. 2015; Phukhamsakda et al. 2016). More recently, the genera *Corylicola*, *Flavosporella*, and *Neopalmiascoma* have been incorporated into Bambusicolaceae (Wijesinghe et al. 2020; Yu et al. 2024; Zhang et al. 2024), bringing the current number of recognized genera to six: *Bambusicola*, *Corylicola*, *Flavosporella*, *Leucaenicola*, *Neopalmiascoma*, and *Palmiascoma*. Among these, *Bambusicola* remains the largest and most extensively studied genus. It is morphologically characterized by uni-to multi-loculate, immersed ascomata, bitunicate, cylindrical asci with a short furcate pedicel and a shallow apical ocular chamber and 2–3-seriate, slightly broad-fusiform, 1-septate hyaline ascospores, surrounded by a gelatinous sheath (Dai et al. 2012, 2017; Hyde et al. 2013; Liu et al. 2015). Its asexual morphs exhibit indistinct conidiophores, holoblastic, annelidic, discrete, cylindrical conidiogenous cells, and pale brown to dark brown, 1–3-septate, cylindrical conidia (Dai et al. 2012, 2017; Hyde et al. 2013; Liu et al. 2015; Jayasiri et al. 2019).

Given the increasing recognition of bambusicolous fungal diversity, our study aimed to explore the microfungi associated with decaying bamboo in Guizhou, China. As a result, we identified five species belonging to Bambusicolaceae. To resolve their taxonomic placement and phylogenetic relationships, we conducted morphological comparisons and multi-gene phylogenetic analyses using a concatenated dataset of SSU, ITS, LSU, *rpb2*, and *tef1-α* sequences. Our findings revealed three novel species: *Bambusicolapseudodimorphae*, *B.gelatinosospora*, and *B.ellipsospora*. Additionally, we have also identified two known species, namely *Bambusicolaautumnalis* and *Corylicolaitalica*. Notably, this study represents the first report of the asexual morph of *B.autumnalis*, and *C.italica* is newly recorded from China. We provide comprehensive morphological descriptions, illustrations, and molecular phylogenetic insights, contributing to the growing understanding of bambusicolous fungi.

## ﻿Materials and methods

### ﻿Sample collection, morphological studies, and isolation

Decayed culms were collected from Guizhou Province, China. Fungal fruiting bodies were examined using a stereomicroscope (Motic SMZ 168). Freehand sections of ascomata and other fungal structures were mounted in water for microscopic studies and photomicrography. Morphological observations were made using a Nikon ECLIPSE Ni compound microscope fitted with a Nikon DS-Ri2 digital camera. All measurements were made with Tarosoft Image FrameWork software (IFW) (Liu et al. 2010). Photo plates were processed with Adobe Photoshop CS6 software (Adobe Systems, USA). Single spore isolations were carried out following the method in Chomnunti et al. (2014). Type specimens were deposited in the herbarium of Cryptogams Kunming Institute of Botany Academia Sinica (HKAS), Kunming, China, and herbarium of Guizhou Academy of Agriculture sciences (GZAAS), Guiyang, China. Pure cultures were deposited in China General Microbiological Culture Collection Center (CGMCC), Beijing, China, and Guizhou Culture Collection (GZCC), Guiyang, China. Facesoffungi (http://www.facesoffungi.org/) numbers were obtained as in Jayasiri et al. (2015). The new species are registered in Index Fungorum (2025, http://www.indexfungorum.org/).

### ﻿DNA extraction, PCR amplification and sequencing

Fungal mycelia were scraped from the pure culture which was growing on PDA (Potato Dextrose Agar) for one week at 25 °C in the dark. The total genomic DNA was extracted using an Ezup Column Fungi Genomic DNA Purification Kit (Sangon Biotech, China) from fresh fungal mycelia. Five gene regions, small subunit rDNA (SSU), internal transcribed spacer (ITS), large subunit rDNA (LSU), RNA polymerase II subunit 2 (*rpb2*) and translation elongation factor 1-alpha (*tef1-α*) were amplified using the primer pairs NS1/NS4 (White et al. 1990), ITS4/ITS5, LR0R/LR5 (Vilgalys and Hester 1990), fRPB2-5f /fRPB2-7cR (Liu et al. 1999) and ef1-983F/ef1-2218R (Rehner and Buckley 2005), respectively. Polymerase chain reaction (PCR) was carried out in a 25 μL reaction volume containing 12.5 μL 2 × PCR Master Mix (Sangon Biotech, China), 9.5 μL ddH_2_O, 1 μL of each primer and 1 μL DNA template. The amplification conditions for SSU, ITS, LSU and *tef1-α* gene regions followed Tennakoon et al. (2016), for *rpb2* gene regions followed Du et al. (2024). PCR products were examined using 1.2% agarose electrophoresis gel stained with ethidium bromide and sequenced by Sangon Biotech (Shanghai) Co., Ltd, China. Newly generated nucleotide sequences were submitted in GenBank (Table 1).

**Table 1.** Isolates used in this study.

Taxa	Vouchers/Strains	GenBank accession numbers				
		SSU	ITS	LSU	*rpb2*	*tef1-α*
*Bambusicolaaquatica*	MFLUCC 18-1031^T^	MT864293	MT627729	MN913710	MT878462	MT954392
*Bambusicolaautumnalis*	CGMCC 3.24280^T^	OQ427823	OQ427824	OQ427825	OQ507621	OQ507622
***Bambusicolaautumnalis***	CGMCC 3.20354	OR853568	OR853544	OR853556	PP084027	PP111465
***Bambusicolaautumnalis***	GZCC 21-0897	OR853569	OR853545	OR853557	PP084028	PP111466
*Bambusicolabambusae*	MFLUCC 11-0614^T^	JX442039	JX442031	JX442035	KP761718	KP761722
*Bambusicoladidymospora*	MFLUCC 10-0557^T^	KU872110	KU940116	KU863105	KU940163	KU940188
*Bambusicoladimorpha*	MFLUCC 13-0282^T^	KY038354	KY026582	KY000661	KY056663	N/A
***Bambusicolaellipsospora***	CGMCC 3.20243**^T^**	OR853572	OR853548	OR853560	PP084031	PP111469
***Bambusicolaellipsospora***	GZCC 21-899	OR853573	OR853549	OR853561	PP084032	PP111470
*Bambusicolaficuum*	MFLUCC 17-0872^T^	MT215581	N/A	MT215580	N/A	MT199326
*Bambusicolafusispora*	MFLUCC 20-0149^T^	MW076529	MW076532	MW076531	MW034589	N/A
***Bambusicolagelatinosospora***	CGMCC 3.20355**^T^**	OR853570	OR853546	OR853558	PP084029	PP111467
***Bambusicolagelatinosospora***	GZCC 21-0898	OR853571	OR853547	OR853559	PP084030	PP111468
*Bambusicolaguttulata*	CGMCC 3.20935^T^	ON332919	ON332909	ON332927	ON383985	ON381177
*Bambusicolahongheensis*	KUN-HKAS 129042^T^	OR501419	OR233600	OR335804	OR540736	N/A
*Bambusicolairregulispora*	MFLUCC 11-0437^T^	JX442040	JX442032	JX442036	KP761719	KP761723
*Bambusicolaloculata*	MFLUCC 13-0856^T^	KP761735	KP761732	KP761729	KP761715	KP761724
*Bambusicolamassarinia*	MFLUCC 11-0389^T^	JX442041	JX442033	JX442037	KP761716	KP761725
*Bambusicolameishanensis*	CGMCC 3.25594^T^	PQ066540	PQ067775	PQ067690	N/A	PQ278548
*Bambusicolananensis*	MFLUCC 21-0063^T^	N/A	OK491656	OK491652	N/A	N/A
***Bambusicolapseudodimorphae***	CGMCC 3.20353**^T^**	OR853566	OR853542	OR853554	PP084025	PP111463
***Bambusicolapseudodimorphae***	GZCC 21-0896	OR853567	OR853543	OR853555	PP084026	PP111464
*Bambusicolapustulata*	MFLUCC 15-0190^T^	KU872112	KU940118	KU863107	KU940165	KU940190
*Bambusicolasichuanensis*	SICAUCC 16-0002^T^	MK253528	MK253473	MK253532	MK262830	MK262828
*Bambusicolasplendida*	MFLUCC 11-0439^T^	JX442042	JX442034	JX442038	KP761717	KP761726
*Bambusicolasubthailandica*	SICAUCC 16-0005^T^	MK253529	MK253474	MK253533	MK262831	MK262829
*Bambusicolathailandica*	MFLUCC 11-0147^T^	N/A	KU940119	KU863108	KU940166	KU940191
*Bambusicolatriseptatispora*	MFLUCC 11-0166^T^	N/A	KU940120	KU863109	KU940167	N/A
*Corylicolaitalica*	MFLUCC 19-0500	MT554923	MT554925	MT554926	MT590776	N/A
*Corylicolaitalica*	MFLUCC 20-0111^T^	MT633084	MT633085	MT626713	MT635596	MT590777
***Corylicolaitalica***	GZCC 21-0757	PQ571780	PQ571751	PQ571778	N/A	PQ591923
*Leucaenicolaaseptata*	MFLUCC 17-2423^T^	MK347853	MK347746	MK347963	MK434891	MK360059
*Leucaenicolacamelliae*	NTUCC 18-093-4^T^	MT071229	MT112302	MT071278	MT743283	MT374091
*Leucaenicolaosmanthi*	NTUCC 18-101-1^T^	MN908609	MN908565	MN908612	MN915020	MN918596
*Leucaenicolaphraeana*	MFLUCC 18-0472^T^	MK347892	N/A	MK348003	MK434867	MK360060
*Leucaenicolataiwanensis*	NTUCC 18-094-1^T^	MT071228	MT112301	MT071277	N/A	N/A
*Neopalmiascomamacadamiae*	ZHKUCC 23-0746^T^	OR978323	OR978321	OR978582	OR983253	OR999080
*Neopalmiascomamacadamiae*	ZHKUCC 23-0747	OR978324	OR978322	OR978583	OR983254	OR999081
*Palmiascomagregariascomum*	MFLUCC 11-0175^T^	KP753958	KP744452	KP744495	KP998466	N/A
*Palmiascomaqujingense*	KUMCC 19-0201^T^	MT477186	MT477183	MT477185	MT495782	N/A
*Sulcatisporaacerina*	KT2982	LC014605	LC014597	LC014610	N/A	LC014615
*Sulcatisporaberchemiae*	KT1607	AB797244	AB809635	AB807534	N/A	AB808509

**Note**: “T” stands for ex-type strains and missing data are shown as “N/A”. Sequences highlighted in bold were generated in this study.

### ﻿Phylogenetic analyses

Analyses of Bambusicolaceae were performed respectively. Both phylogenetic analyses were performed based on SSU, ITS, LSU, *rpb2* and *tef1-α* sequence data. The representative strains of Bambusicolaceae (Table 1) were referred to BLAST (https://blast.ncbi.nlm.nih.gov/Blast.cgi) results and relevant publications (Yu et al. 2022, 2024; Liang et al. 2023; Zhang et al. 2024). Sequences were aligned using MAFFT v. 7 (Katoh and Standley 2013). Manual adjustments were performed when necessary using BioEdit v. 7.0 (Hall 1999). Phylogenetic analyses of maximum likelihood (ML) and bayesian inference (BI) were carried out as detailed in Dissanayake et al. (2020) and performed with raxmlGUI v. 1.3 (Silvestro and Michalak 2012) and MrBayes v3.1. (Huelsenbeck and Ronquist 2001; Zhaxybayeva and Gogarten 2002; Nylander 2004). Phylogenetic trees were visualized with FigTree v1.4.2 (Rambaut 2012) and edited using Adobe Illustrator 2021 (2.6.0.44) and Adobe Photoshop CS6 software (Adobe Systems, USA).

## ﻿Results

### ﻿Phylogeny

**Figure 1.**
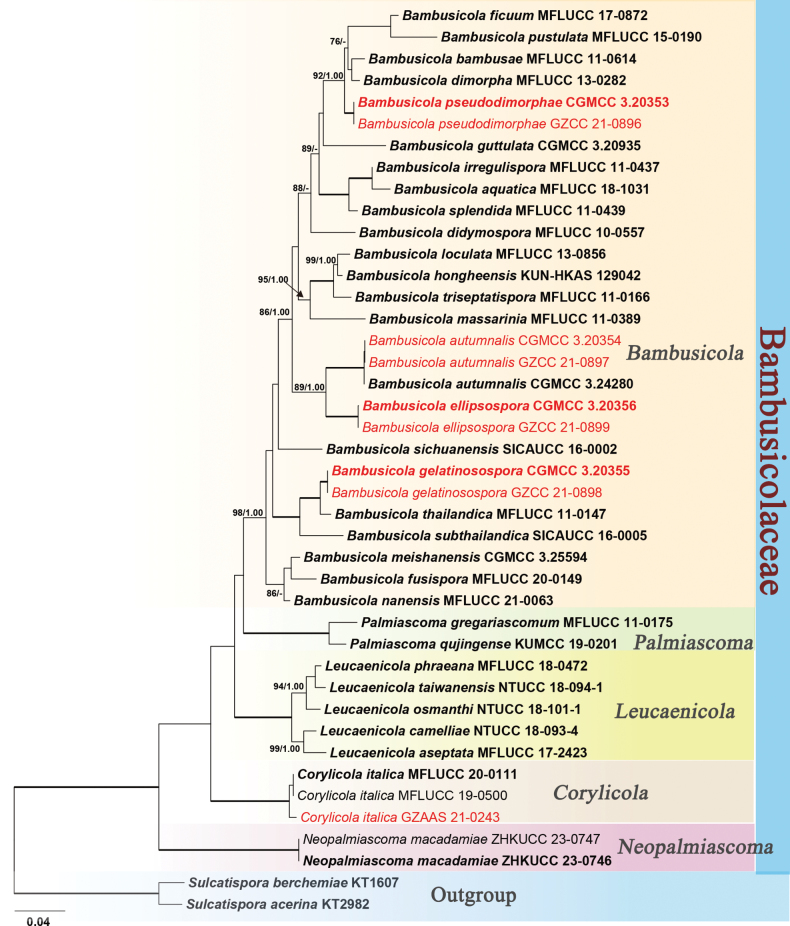
Phylogram generated from maximum likelihood analysis based on a combined dataset of SSU, ITS, LSU, *rpb2* and *tef1-α* sequences. Bootstrap support values for ML (≥75%) and bayesian posterior probabilities (≥0.95) are given at the nodes (ML BS/PP). Branches with 100% ML BS and 1.00 PP are thickened. New strains are shown in red. The tree is rooted with *Sulcatisporaberchemiae* KT1607 and *Sulcatisporaacerina* KT2982, and ex-type strains are in bold.

To determine the phylogenetic placements of the new collections in this study, the combined SSU, ITS, LSU, *rpb2* and *tef1-α* data sets, comprising 42 taxa with *Sulcatisporaberchemiae* KT1607 and *Sulcatisporaacerina* KT2982 as the outgroup taxa. The dataset comprises 4, 641 characters (SSU: 1-1001; ITS: 1002-1896; LSU: 1897-2701; *rpb2*: 2702-3691; *tef1-α*: 3692-4641) after alignment, including gaps. Maximum likelihood and bayesian analyses were performed, respectively, and both methods yielded consistent topologies. The best-scoring RAxML tree (Fig. 1) was obtained with a final likelihood value of -23443.659740. Estimated base frequencies were as follows: A = 0.243090, C = 0.252645, G = 0.271896, T = 0.228350; substitution rates AC = 1.173578, AG = 2.804498, AT = 0.917016, CG = 1.080920, CT = 5.912507, GT = 1.000000; The gamma distribution shape parameter alpha is equal to 0.305487 and the Tree-Length equal to 0.166660.

Phylogenetic analyses showed that the nine newly obtained strains of Bambusicolaceae clustered into five clades and can be recognized as three new species (*Bambusicolaellipsospora*, *B.gelatinosospora*, *B.pseudodimorphae*) and two known species (*B.autumnalis*, *Corylicolaitalica*) (Fig. 1).

## ﻿Taxonomy

### ﻿ Bambusicola ellipsospora

Taxon classificationFungiPleosporalesBambusicolaceae

Y. Feng, Z.Y. Liu & Jian K. Liu sp. nov.

DFB58706-5BD2-5D60-8A27-987323760EF0

Index Fungorum: IF903223

Facesoffungi Number: FoF15907

Fig. 2

#### Holotype.

HKAS 112592.

#### Etymology.

The epithet refers to the ellipsoidal ascospores.

#### Description.

***Saprobic*** on dead bamboo culms. **Sexual morph**: ***Ascomata*** 215–363 μm diam., 180–288 μm high, solitary, scattered to gregarious, immersed to erumpent, later becoming superficial, forming brown to dark brown, lenticular spots on the host surface with a slit. ***Peridium*** comprising several layers of cells of ***textura angularis***, inner layers comprising hyaline to dark brown, outer layers composed of thick, dark brown to black cells. ***Hamathecium*** of dense, 0.5–1.8 μm wide, filamentous, branched, septate, smooth-walled, trabecular pseudoparaphyses, anastomosing at the apex, embedded in a hyaline, gelatinous matrix. ***Asci*** 70–92 × 15–21 μm (x̄ = 82 × 17 μm, n = 20), 8-spored, bitunicate, fissitunicate, broadly cylindrical to cylindri-clavate, with a short pedicel, apically rounded with a well-developed ocular chamber. ***Ascospores*** (18–)19–24(–25) × (5–)6–8(–9) μm (x̄ = 22 × 7 μm, n = 30), overlapping, biseriate, hyaline, straight, ellipsoidal to fusiform, with obtuse ends, 1-septate, constricted at the septum, smooth-walled with 4 large guttules, surrounded by a thin-gelatinous sheath. **Asexual morph**: Undetermined.

**Figure 2.**
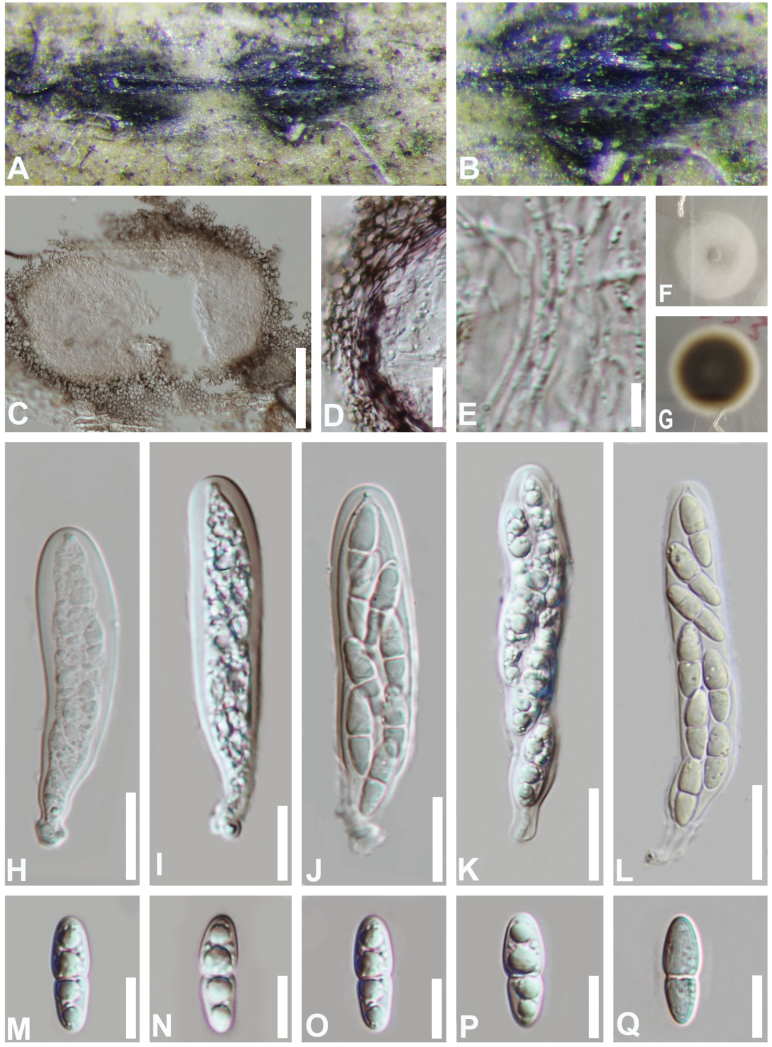
*Bambusicolaellipsospora* (HKAS 112592, holotype) **A, B** appearance of ascomata on the host **C** vertical section of an ascoma **D** peridium **E** pseudoparaphyses **F, G** cultures on PDA**F** from above **G** from below **H–L** asci **M–Q** ascospores. Scale bars: 100 µm (**C**); 10 µm (**D, E, M–Q**); 20 µm (**H–L**).

#### Culture characteristics.

Ascospores germinating on PDA within 12 h. Colonies on PDA slow growing, 13 mm diam. after 10 d at 25 °C in the dark, circular, white, velvety, with dense mycelium on the surface; in reverse brown in the center, becoming paler to white at the entire margin.

#### Material examined.

China • Guizhou Province, Chishui City. On dead culms of bamboo, 10 July 2019, Yao Feng CS018 (HKAS 112592, holotype; GZAAS 21-0503, isotype), ex-type living cultures CGMCC 3.20356 = GZCC 21-0803; *ibid*., CS036 (GZAAS 21-0391, paratype), living culture GZCC 21-899.

#### Notes.

Phylogenetic analyses revealed that the two strains of *Bambusicolaellipsospora* (CGMCC 3.20356 and GZCC 21-0899) formed a distinct clade, sister to *B.autumnalis* (CGMCC 3.24280, CGMCC 3.20354, GZCC 21-0897) (Fig. 1). *Bambusicolaellipsospora* can be distinguished from *B.autumnalis* based on sequence divergence in *tef1-α* (874/927 bp) and *rpb2* (699/784 bp). Additionally, morphological differences further support their distinction, as *B.ellipsospora* produces ellipsoidal to fusiform ascospores with obtuse rounded ends, while *B.autumnalis* has fusiform and slightly curved ascospores with narrower acute ends (Liang et al. 2023). Moreover, the ascospore of *B.ellipsospora* collected in this study shows distinct constriction at the septum, whereas that of *B.autumnalis* only has slight constriction. Based on morphological and phylogenetic evidence, *B.ellipsospora* is recognized as a novel species.

### ﻿ Bambusicola gelatinosospora

Taxon classificationFungiPleosporalesBambusicolaceae

Y. Feng, Z.Y. Liu & Jian K. Liu sp. nov.

35975FA1-8AEE-5A35-8D46-F6C6ABB14D31

Index Fungorum: IF903224

Facesoffungi Number: FoF15908

Fig. 3

#### Holotype.

HKAS 112599.

#### Etymology.

The epithet refers to the gelatinous sheath of ascospores.

#### Description.

***Saprobic*** on dead bamboo culms, forming raised, black spots on the host surface. **Sexual morph**: ***Ascomata*** 320–345 μm diam, 170–230 μm high, solitary, scattered to gregarious, immersed, subglobose with a flattened base. ***Peridium*** comprising several layers of cells of *textura angularis*, less distinguished from the host tissue. ***Hamathecium*** of dense, 1–2 μm wide, filamentous, branched, septate, smooth-walled, trabecular pseudoparaphyses, anastomosing at the apex, embedded in a hyaline, gelatinous matrix. ***Asci*** 60–90 × 10–12 μm (x̄ = 77 × 11 μm, n = 20), 8-spored, bitunicate, fissitunicate, broadly cylindrical to cylindrical-clavate, with a short pedicel, apically rounded with a well-developed ocular chamber. ***Ascospores*** (16–)20–25 × 4–6 μm (x̄ = 22 × 5 μm, n = 30), overlapping, biseriate, hyaline, fusiform, slightly bent with acute ends, 3-septate, slightly constricted at the septa, smooth-walled, guttulate, surrounded by a gelatinous sheath. **Asexual morph**: Undetermined.

**Figure 3.**
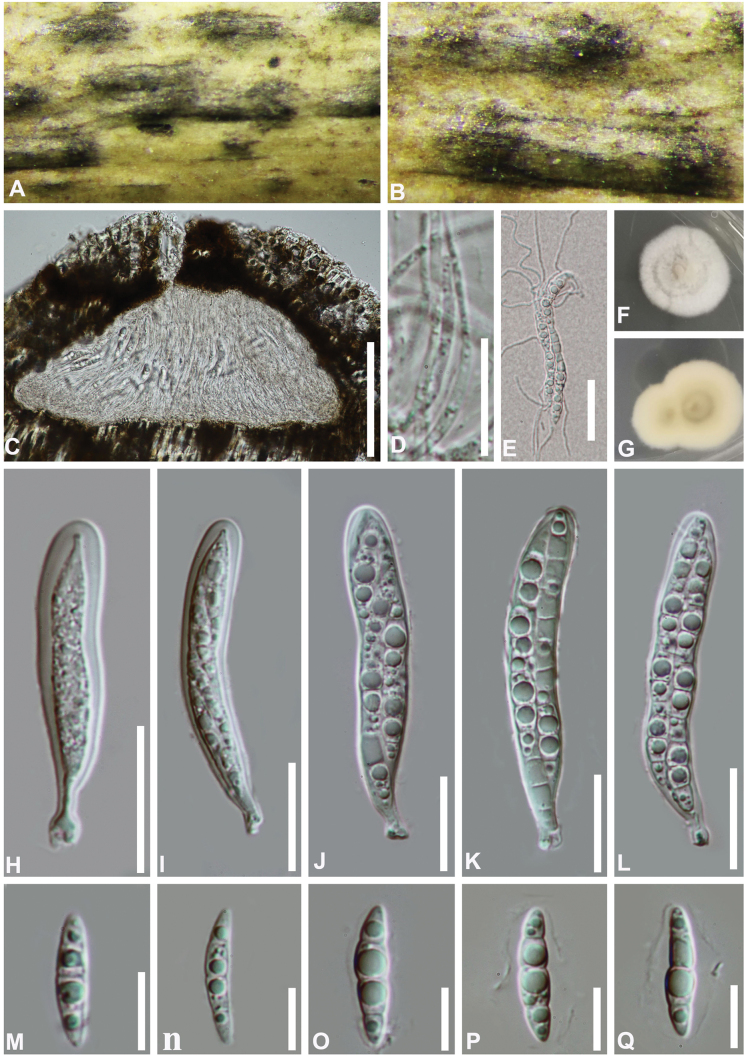
*Bambusicolagelatinosospora* (HKAS 112599, holotype) **A, B** appearance of ascomata on the host **C** vertical section of an ascoma **D** pseudoparaphyses **E** germinated ascospores **F, G** cultures on PDA**F** from above **G** from below **H–L** asci **M–Q** ascospores. Scale bars:100 µm (**C**); 30 µm (**E**); 20 µm (**H–L**); 15 µm (**D, M–Q**).

#### Culture characteristics.

Colonies on PDA slow growing, reaching 15 mm diam. after 10 d at 25 °C in dark. circular, felted, with white dense mycelium on the surface. In reverse pale yellow, becoming brown from the center, with an entire margin.

#### Material examined.

China • Guizhou Province, Xingyi City. On dead stem of bamboo, 5 September 2019, Yao Feng, XY19-11 (HKAS 112599, holotype; GZAAS 21-0502, isotype), ex-type living cultures CGMCC 3.20355 = GZCC 21-0802; *ibid*., on dead branches on bamboo, 5 September 2019, Yao Feng, XY19a (GZAAS 21-0390; paratype), living cultures GZCC21-0898.

#### Notes.

Phylogenetic analyses revealed that the two strains of *Bambusicolagelatinosospora* (CGMCC 3.20355 and GZCC 21-0898) clustered with *B.thailandica* (MFLUCC 11-0147) and *B.subthailandica* (SICAUCC 16-0005) as a distinct lineage (Fig. 1). Morphologically, *B.gelatinosospora* closely resembles *B.thailandica* and *B.subthailandica*, sharing key characteristics such as ostiolate ascomata, cylindrical to cylindri-clavate asci, and hyaline, fusiform, 3-septate ascospores with acute ends (Dai et al. 2017; Yang et al. 2019). Although these species are morphologically indistinguishable, their cultures exhibit differences in colors and colony morphology (Table 2). Additionally, they can be differentiated by their minimal sequence similarities. In a comparison of ITS, LSU, *rpb2* and *tef1-α* nucleotides, *B.gelatinosospora* (CGMCC 3.20355) has 98%, 99%, 97% and 99% similarity, in ITS (702/713 bp, 1 gap), LSU (817/819 bp, no gaps), *rpb2* (949/974 bp, no gaps), and *tef1-α* (914/920 bp, no gap), which is different from *B.thailandica* (MFLUCC 11-0147). These findings combined with its morphological and molecular distinctiveness strongly supported the conclusion that *Bambusicolagelatinosospora* represents a new species within the genus *Bambusicola*.

**Table 2.** Morphological comparisons of *Bambusicolagelatinosospora* and similar taxa within Bambusicolaceae.

Morphological characteristics	*Bambusicolagelatinosospora* (In this study)	*Bambusicolathailandica* (Dai et al. 2017)	*Bambusicolasubthailandica* (Yang et al. 2019)
Ascomata	320–345 μm diam.; 170–230 μm high	310–400 μm diam.; 90–155 μm high	326–377 μm diam.; 226–277 μm high
Asci	60–90 × 10–12 μm; (x̄ = 77 × 11 μm, n = 20)	(60–)70– 90(−97) × 10–12(−14) μm; (x̄ = 75.5 × 12 μm, n = 20)	(48–)65–86(–119) × (7.5–)9–12 μm; (x̄ = 75.6 × 11 μm, n = 40)
Ascospores	(16–)20–25 × 4–6 μm; (x̄ = 22 × 5 μm, n = 30)	(16–)18– 22(−24) × (3.5–)4–6 μm; (x̄ = 2 0.8 × 5 μm, n = 30)	(20–)21–24(–26) × (4–)5–6(–6.5) μm; (x̄ = 22.6 × 5.7 μm, n = 50)
Culture colour	white, reverse pale yellow	white to cream	grey to grey brown
Culture texture	felted, with white dense mycelium on the surface	fluffy to floccose	fluffy

### ﻿ Bambusicola pseudodimorphae

Taxon classificationFungiPleosporalesBambusicolaceae

Y. Feng, Z.Y. Liu & Jian K. Liu sp. nov.

AB9367A4-1559-5985-A9C9-E75214A6AA8B

Index Fungorum: IF903225

Facesoffungi Number: FoF15909

Figs 4 , 5

#### Holotype.

HKAS 112597.

#### Etymology.

Species epithet refers to the similar conidia to *Bambusicoladimorpha* which have two types in culture.

#### Description.

***Saprobic*** on bamboo culms. **Sexual morph**: Undetermined. **Asexual morph**: ***Conidiomata*** initially embedded in the epidermis of bamboo culms and later breaking through the epidermis,solitary to gregarious, subglobose with a flattened base, brown to dark brown. 78–161 μm high, 180–270 μm diam. ***Conidiomatal wall*** comprising 3–5 layers of cells of ***textura angularis***, with dark brown outer layers and hyaline inner layers, less distinguished from the host tissue. ***Conidiophores*** reduced to conidiogenous cells. ***Conidiogenous cells*** 9–32 × 1–2 μm (x̄ = 23 × 2 μm, n = 15), holoblastic, monoblastic, cylindrical, smooth, hyaline. ***Conidia*** 24–27 × 3–4 μm (x̄ = 26 × 4 μm, n = 30), initially hyaline to pale brown, aseptate, becoming yellowish brown, 2–3-septate when maturity, cylindrical, obtuse at both ends, smooth-walled, straight, guttulate.

**Figure 4.**
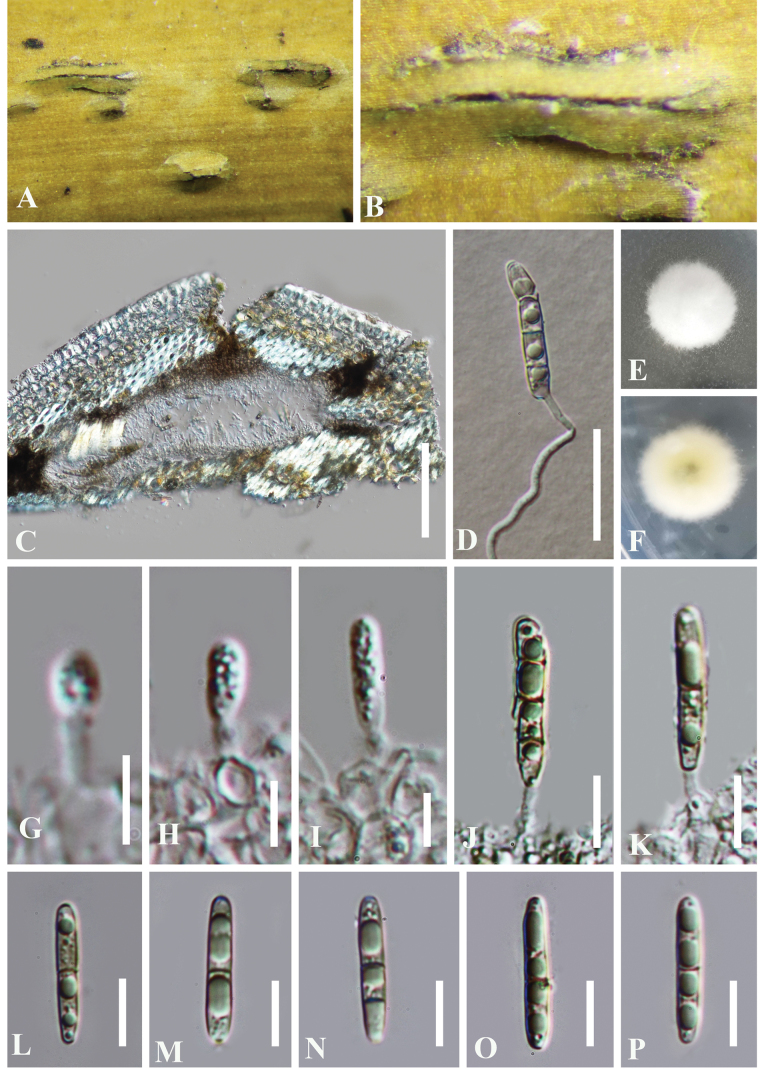
*Bambusicolapseudodimorphae* (HKAS 112597, holotype) **A, B** conidiomata on the host **C** vertical section of a conidioma **D** germinated conidium **E, F** cultures on PDA**E** from above **F** from below **G−K** conidiogenous cells and developing conidia **L−P** conidia. Scale bars: 100 µm (**C**); 20 µm (**D**); 5 µm (**G−I**); 10 µm (**J−P**).

#### Culture characteristics.

Colonies on PDA slow growing, 16 mm diam. after 10 d at 25 °C in dark, circular, with an entire margin, white, fluffy, with dense mycelium on the surface. Reverse white with a yellowish center. After being cultivated on PDA for two months, *Bambusicolapseudodimorphae* generated two types of conidia, macro- and microconidia. Macroconidia 20–26(–29) × 3–5 μm (x̄ = 24 × 4 μm, n = 30), cylindrical, with obtuse and rounded ends, 3-septate, pale brown to brown, smooth-walled. Microconidia 3–4 × 1–3 μm (x̄ = 4 × 2 μm, n = 30), ellipsoidal, pale brown, aseptate.

**Figure 5.**
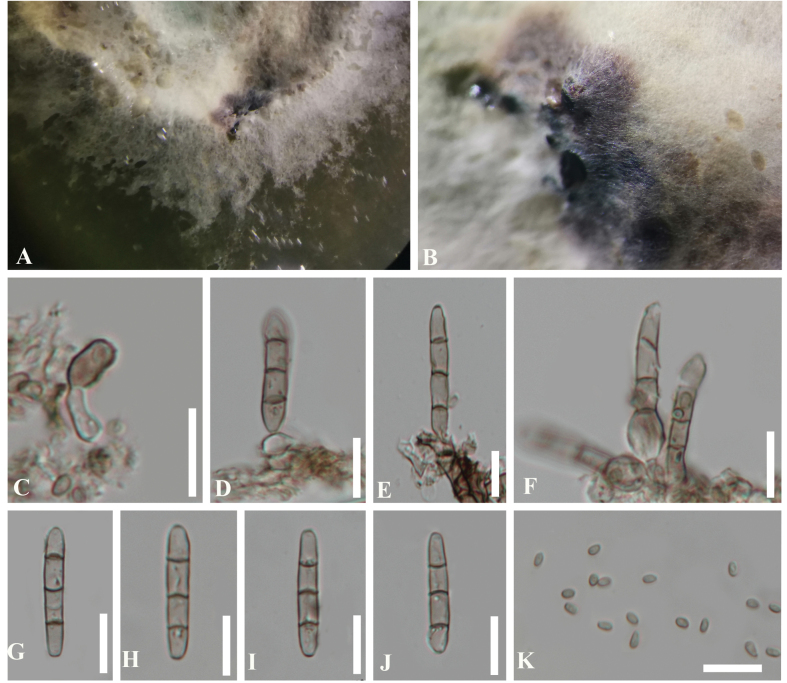
Reproduced *Bambusicolapseudodimorphae* (CGMCC 3.20353, ex-type) **A, B** colony on PDA**C−F** conidiogenous cells with conidia **G−J** macroconidia **K** microconidia. Scale bars: 10 µm (**C−K**).

#### Material examined.

China • Guizhou Province, Xingyi City. On dead bamboo stems, 5 September 2019, Yao Feng, XY19-3 (HKAS 112597, holotype; GZAAS 21-0501, isotype), ex-type living cultures CGMCC 3.20353 = GZCC21-0801; *ibid*., 6 September 2019, Yao Feng, XY19b (GZAAS 21-0388, paratype), living culture GZCC 21-0896.

#### Notes.

Phylogenetic analyses revealed that the two strains of *Bambusicolapseudodimorphae* clustered with *B.bambusae*, *B.dimorpha*, *B.ficuum*, and *B.pustulata*, forming a distinct lineage within *Bambusicola* (Fig. 1). Morphologically, *B.pseudodimorphae* closely resembles is similar to *B.dimorpha* in having two types of conidia. However, *B.pseudodimorphae* differs by having smaller conidiomata (78–161 × 180–270 μm vs. 200–250 × 210–340 μm) and longer conidia (24–27 μm long, 3-septate vs. 13–21 μm long, 1-septate) (Thambugala et al. 2017). Since our collection is an asexual morph, and the other three species (*B.bambusae*, *B.ficuum*, and *B.pustulata*) are known as sexual morphs, direct comparisons between them are not feasible. Molecular analyses further support the distinction of *B.pseudodimorphae* as a novel species. The *tef1-α* and *rpb2* sequences of *B.pseudodimorphae* differ from those of *B.bambusae* by 3% (32/924 bp) and 3% (35/1001 bp) nucleotides, respectively. Additionally, the *rpb2* sequence of *B.pseudodimorphae* showed 96% (945/984 bp, one gap) and 97% (853/880 bp, no gap) sequence similarity with *B.pustulata* and *B.dimorpha*, respectively. Based on the morphology and molecular evidence, *B.pseudodimorphae* is identified as a new species following the species delineation guidelines of Jeewon and Hyde (2016).

### ﻿ Bambusicola autumnalis

Taxon classificationFungiPleosporalesBambusicolaceae

R.R. Liang, S.N. Zhang & Jian K. Liu, Phytotaxa 601(3): 208 (2023)

94562EFE-AD97-5611-A9EB-0A00DBC9F8DF

Figs 6 , 7

#### Description.

***Saprobic*** on dead bamboo culms. **Sexual morph: *Ascomata*** 263–370 μm diam., 185–202 μm high, solitary, scattered to gregarious, initially growing under the host surface, breaking through the surface after maturity, with cracks on the host, subglobose with a flattened base, some arranged in rows, brown to dark brown. ***Peridium*** comprising several layers of cells of ***textura angularis***, with dark brown outer layers and hyaline inner layers. ***Hamathecium*** of dense, 1–2 μm wide, filamentous, branched, indistinctly septate, smooth-walled, trabeculate pseudoparaphyses, anastomosing at the apex, embedded in a hyaline, gelatinous matrix. ***Asci*** 64–95(−102) × (10–)11−14 μm (x̄ = 83 × 12 μm, n = 20), 8-spored, bitunicate, broadly cylindrical to cylindrical-clavate, with a short pedicel, apically rounded with a well-developed ocular chamber. ***Ascospores*** (24–)26–29 × 4–6 μm (x̄ = 26 × 5 μm, n = 30), overlapping, biseriate, hyaline, fusiform, with acute ends, 1-septate, slightly constricted at the septa, asymmetric, slightly curved, smooth-walled with 2–4 guttules, surrounded by a thin, inconspicuous mucilaginous sheath. **Asexual morph**: Coelomycetous, produced on PDA. ***Conidiomata*** pycnidial, solitary to gregarious, superficial. ***Conidiogenous cells*** 5–17 × 2–4 μm (x̄ = 9 × 3 μm, n = 15), holoblastic, integrated or discrete, subcylindrical to cylindrical, hyaline, smooth, occasionally with an enlarged structure at the top. ***Conidia*** with two types, macro- and microconidia. ***Macroconidia*** 23–31(–34) × 4–6(–7) μm (x̄ = 28 × 5 μm, n = 30), cylindrical to ellipsoidal, with rounded ends, 3-septate, pale brown to brown, slightly constricted at the septa, smooth. ***Microconidia*** 3–6 × 1–2(–3) μm (x̄ = 5 × 2 μm, n = 30), subglobose to ellipsoidal, with rounded to obtuse ends, pale brown, aseptate, smooth-walled.

**Figure 6.**
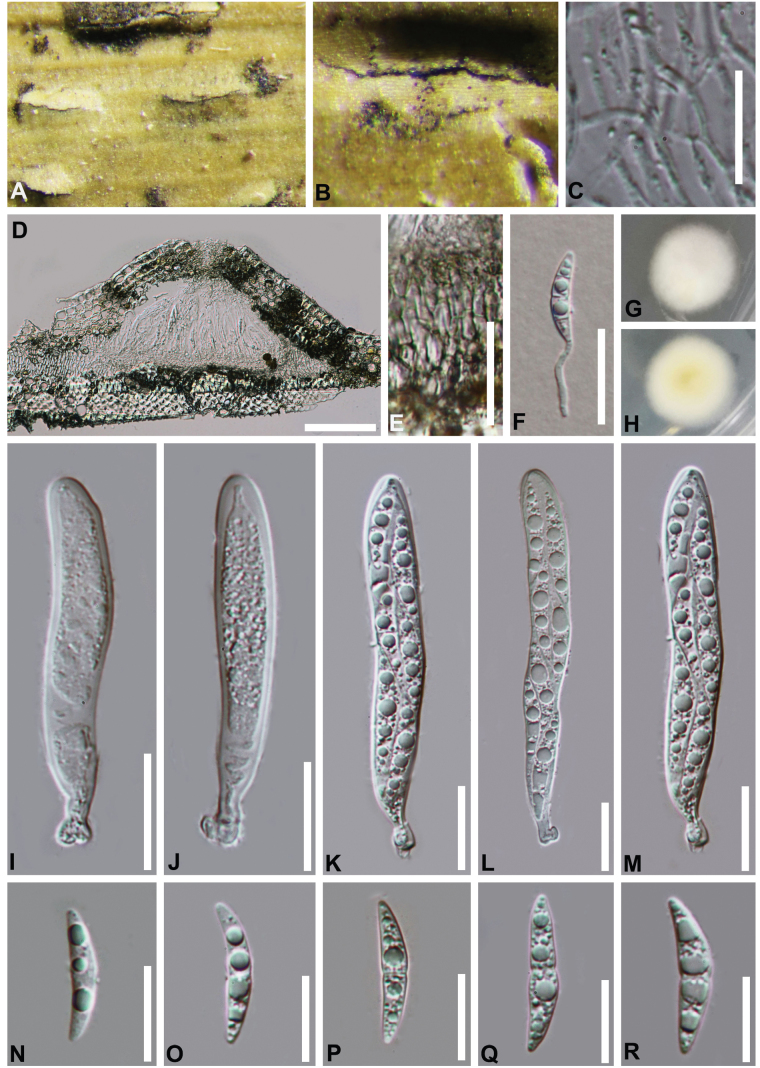
*Bambusicolaautumnalis* (HKAS 112598) **A, B** appearance of ascomata on the host **C** pseudoparaphyses **D** vertical section of an ascoma **E** peridium **F** germinated ascospore **G, H** cultures on PDA**G** from above **H** from below **I–M** asci **N–R** ascospores. Scale bars: 100 µm (**D**); 25 µm (**E, F**); 20 µm (**I–M**); 15 µm (**C, N–R**).

#### Culture characteristics.

Colonies on PDA reaching 16 mm diam. after 10 d at 25 °C in dark, white, circular, velvety, with uneven margin. Reverse white with a yellowish center.

#### Material examined.

China • Guizhou Province, Xingyi City. On dead bamboo stems, 5 September 2019, Yao Feng, XY19-4 (HKAS 112598,GZAAS 21-0500, living cultures CGMCC 3.20354 = GZCC 21-0800; *ibid*., 6 September 2019, Yao Feng, XY19-1 (GZAAS 21-0389), living culture GZCC 21-0897.

**Figure 7.**
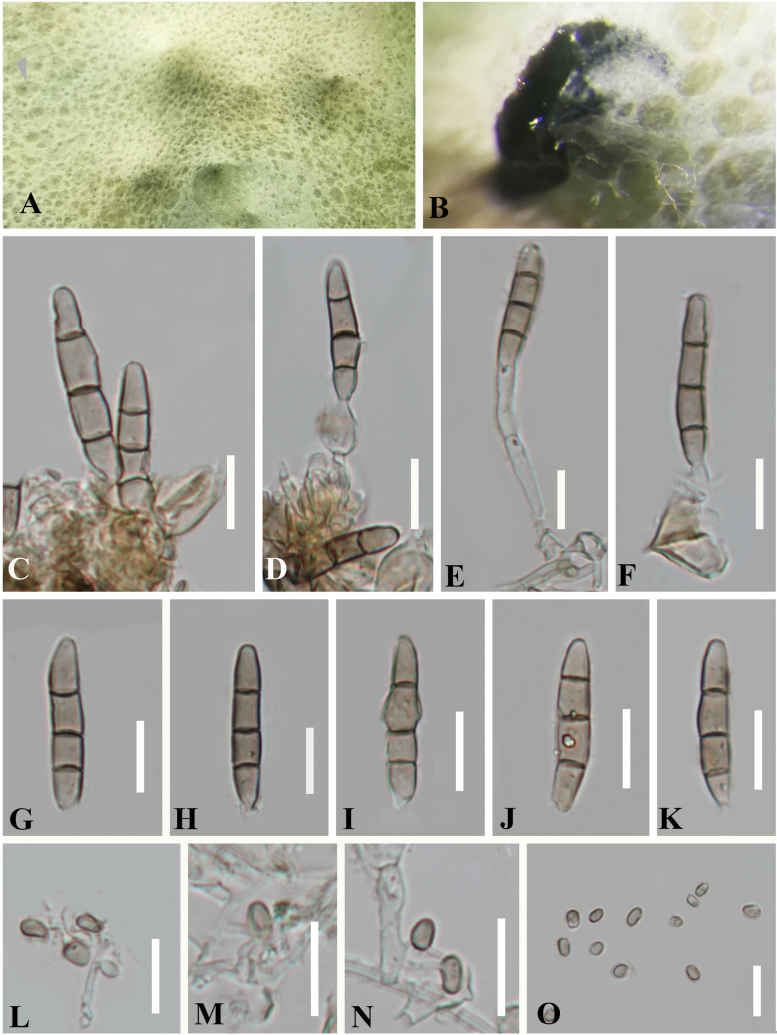
Reproduced *Bambusicolaautumnalis* (CGMCC 3.20354) **A, B** colony on PDA**C−F** conidiogenous cells with macroconidia **G−K** macroconidia **L−N** conidiogenous cells with microconidia **O** microconidia. Scale bars: 10 µm (**C−O**).

#### Notes.

Liang et al. (2023) described *Bambusicolaautumnalis* as a novel species based on its sexual morph and phylogenetic analysis. The phylogenetic results (Fig. 1) confirmed that our new collections are conspecific with *B.autumnalis*, and this study expands upon their findings by providing a detailed description of the asexual morph, thereby enhancing the taxonomic understanding of this species.

### ﻿ Corylicola italica

Taxon classificationFungiPleosporalesBambusicolaceae

Wijesinghe, Camporesi, Yong Wang bis & K.D. Hyde, Biodiversity Data Journal 8 (e55957): 8 (2020)

ED4AE79B-9061-5BFA-9B15-8B1973B7873F

Fig. 8

#### Description.

***Saprobic*** on the branches of *Prunusserrulata*. **Sexual morph**: ***Ascomata*** 186–292 µm high, 179–267 µm diam., solitary, scattered to gregarious, immersed or erumpent, uniloculate with an ostiole. ***Peridium*** 15–30 µm, comprising several layers of cells of ***textura angularis***, with dark brown outer layers and hyaline inner layers. ***Hamathecium*** of dense, 1–2 μm wide, filamentous, branched, septate, smooth-walled, cellular pseudoparaphyses, anastomosing at the apex, embedded in a hyaline, gelatinous matrix. ***Asci*** 53–66 × 6–8 µm (x̄ = 61 × 7 µm, n = 20), 8-spored, bitunicate, fissitunicate, broadly cylindrical to cylindrical-clavate, with a short pedicel, apically rounded. ***Ascospores*** 8–17 × 3–4 µm (x̄ = 13 × 4 µm, n = 30), overlapping, hyaline, fusiform, broader at the top and narrower at the bottom, with a septum in the middle that is deeply constricted, translucent to light brown or brown, rough. **Asexual morph**: Undetermined.

**Figure 8.**
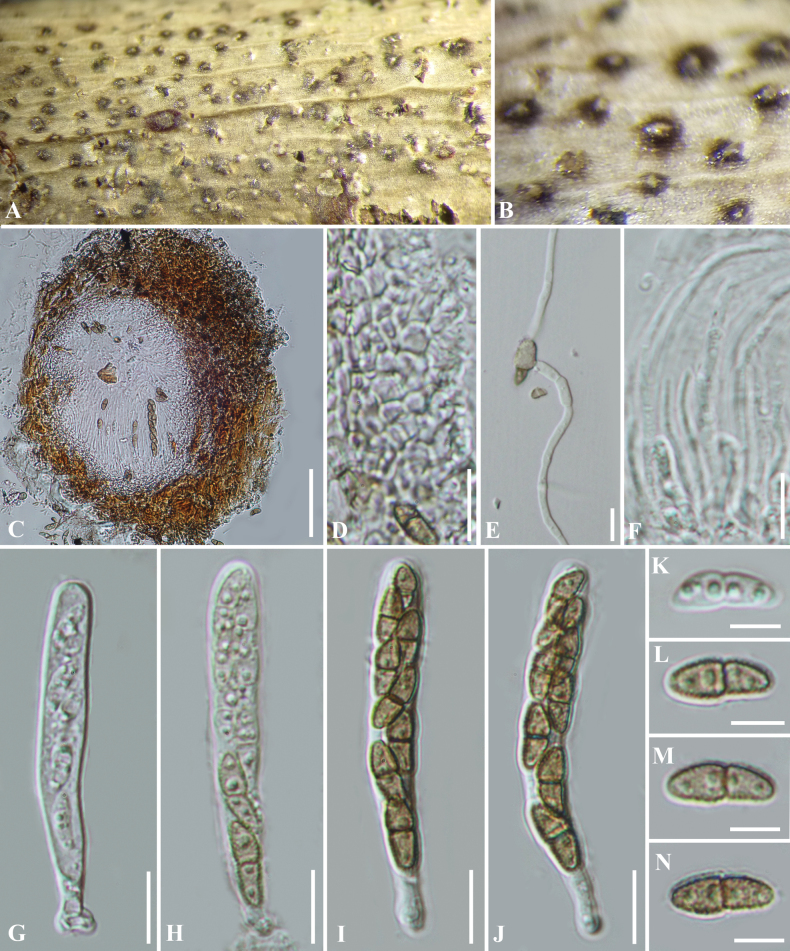
*Corylicolaitalica* (GZAAS 21-0243) **A, B** appearance of ascomata on host **C** vertical section of an ascoma **D** peridium **E** germinated ascospore **F** pseudoparaphyses **G−J** asci **K−N** ascospores. Scale bars: 50 µm (**C**); 10 µm (**D−J**); 5 µm (**K−N**).

#### Culture characteristics.

Ascospores germinating on PDA within 12 h. Colonies on PDA slow-growing, 9 mm diam. after 10 d at 25 °C in the dark, circular, irregular edges, with dense grayish white mycelium on the surface, in reverse brown to black.

#### Material examined.

China • Guizhou Province, Xingyi City. On dead stem of *Prunusserrulata*, 10 July 2018, Yao Feng, nky 141 (GZAAS 21-0243), living culture GZCC 21-0757.

#### Notes.

The morphology and phylogenetic results confirm that our new collections are identical to *Corylicolaitalica*. The genus *Corylicola* was established by Wijesinghe et al. (2020) with *C.italica* as the type species. This species was originally described from a hanging branch of *Corylusavellana* in Italy (Wijesinghe et al. 2020). This study represents the first record of *C.italica* in China.

## ﻿Discussion

This study presents a comprehensive taxonomic investigation of newly isolated fungal species associated with bamboo, contributing to the growing knowledge of fungal diversity within this unique ecological niche. We describe two new sexual morphs, *Bambusicolaellipsospora* and *B.gelatinosospora*, as well as a new asexual morph, *B.pseudodimorphae*. Additionally, we provide a holomorphic description of *B.autumnalis* and report *Corylicolaitalica* for the first time in China. Within the genus *Bambusicola*, eleven species have been described based on their sexual morphs, including *B.aquatica*, *B.autumnalis*, *B.bambusae*, *B.ficuum*, *B.fusispora*, *B.hongheensis*, *B.loculata*, *B.pustulata*, *B.subthailandica*, *B.thailandica*, and *B.meishanensis*. In contrast, five species, *B.guttulata*, *B.irregulispora*, *B.nanensis*, *B.sichuanensis*, and *B.splendida*, are classified based solely on their asexual morphs (Dai et al. 2015; Yang et al. 2019; Brahmanage et al. 2020; Monkai et al. 2021; Phukhamsakda et al. 2022; Yu et al. 2022, 2024; Liang et al. 2023). Notably, holomorph are only known for four species: *B.didymospora*, *B.dimorpha*, *B.massarinia*, and *B.triseptatispora* (Dai et al. 2012, 2017; Thambugala et al. 2017). An intriguing observation in *Bambusicola* is the ability of certain species, such as *B.dimorpha*, *B.nanensis*, *B.sichuanensis*, and *B.pseudodimorphae*, to produce both macroconidia and microconidia in culture (Thambugala et al. 2017; Yang et al. 2019; Phukhamsakda et al. 2022; this study). This phenomenon may be linked to environmental factors, particularly nutrient depletion in the growth medium, which could induce the production of conidia in different forms as an adaptive response. Further studies are required to elucidate the genetic and physiological mechanisms regulating this morphological plasticity.

Currently, *Bambusicola* comprises 20 species (Species Fungorum, accessed 12 February 2025), with 19 species reported from dead bamboo (Dai et al. 2012, 2015, 2017; Dong et al.2020; Thambugala et al. 2017; Monkai et al. 2021; Yang et al. 2019; Yu et al. 2022, 2024, Liang et al. 2023) and only *Bambusicolaficuum* identified from *Ficus* (Boonmee et al. 2021). Examining of species distributions within the genus suggests a strong host preference, with most species exhibiting specificity for bamboo (Dai et al. 2012, 2015, 2017; Dong et al.2020; Thambugala et al. 2017; Yang et al. 2019; Monkai et al. 2021; Yu et al. 2022, 2024; Liang et al. 2023). This raises important ecological questions: Do *Bambusicola* species establish specialized relationships with bamboo through physiological and biochemical mechanisms? Furthermore, does this host specificity make them particularly vulnerable to environmental changes such as climate fluctuations, pest and disease outbreaks, or habitat destruction? Understanding the ecological roles of *Bambusicola* species is essential for assessing their contributions to bamboo ecosystems. Future research should focus on the mechanisms underlying their interactions with bamboo, including potential mutualistic or pathogenic associations. Such studies will contribute to taxonomic resolution and provide insights into conservation strategies to preserve fungal diversity and maintain ecosystem stability.

## Supplementary Material

XML Treatment for Bambusicola ellipsospora

XML Treatment for Bambusicola gelatinosospora

XML Treatment for Bambusicola pseudodimorphae

XML Treatment for Bambusicola autumnalis

XML Treatment for Corylicola italica

## ﻿Additional information

### Author ORCIDs

Yao Feng https://orcid.org/0000-0002-0888-8775

Ya-Ya Chen https://orcid.org/0000-0002-8293-168X

Chuan-Gen Lin https://orcid.org/0000-0003-2750-8720

Zuo-Yi Liu https://orcid.org/0000-0001-5348-8458

Xiao-Fang Chen https://orcid.org/0009-0000-9962-7644

Guo-Shun Pei https://orcid.org/0009-0001-0363-7539

Jian-Kui Liu https://orcid.org/0000-0002-9232-228X

### Data availability

All of the data that support the findings of this study are available in the main text.
